# Discovery of Small Molecule Glycolytic Stimulants for Enhanced ApoE Lipidation in Alzheimer’s Disease Cell Model

**DOI:** 10.3390/ph17040491

**Published:** 2024-04-12

**Authors:** Sachin P. Patil, Bella R. Kuehn

**Affiliations:** 1 NanoBio Lab, Widener University, Chester, PA 19013, USA; 2 Department of Chemical Engineering, Widener University, Chester, PA 19013, USA; brkuehn@widener.edu

**Keywords:** Alzheimer’s disease, astrocytes, glycolysis, liver X receptor (LXR), ABCA1, ABCG1, apolipoprotein E, lipidation

## Abstract

Alzheimer’s disease (AD) is a progressive neurodegenerative disease characterized by pathophysiological deposits of extracellular amyloid beta (Aβ) peptides and intracellular neurofibrillary tangles of tau. The central role of Aβ in AD pathology is well-established, with its increased deposition attributed mainly to its decreased cerebral clearance. Here, it is noteworthy that apolipoprotein E (ApoE), the most significant risk factor for AD, has been shown to play an isoform-specific role in clearing Aβ deposits (ApoE2 > ApoE3 > ApoE4), owing mainly to its lipidation status. In addition to the pathophysiological Aβ deposits, AD is also characterized by abnormal glucose metabolism, which is a distinct event preceding Aβ deposition. The present study established, for the first time, a possible link between these two major AD etiologies, with glucose metabolism directly influencing ApoE lipidation and its secretion by astrocytes expressing human ApoE4. Specifically, glucose dose-dependently activated liver X receptor (LXR), leading to elevated ABCA1 and ABCG1 protein levels and enhanced ApoE lipidation. Moreover, co-treatment with a glycolytic inhibitor significantly inhibited this LXR activation and subsequent ApoE lipidation, further supporting a central role of glucose metabolism in LXR activation leading to enhanced ApoE lipidation, which may help against AD through potential Aβ clearance. Therefore, we hypothesized that pharmacological agents that can target cellular energy metabolism, specifically aerobic glycolysis, may hold significant therapeutic potential against AD. In this context, the present study also led to the discovery of novel, small-molecule stimulants of astrocytic glucose metabolism, leading to significantly enhanced lipidation status of ApoE4 in astrocytic cells. Three such newly discovered compounds (lonidamine, phenformin, and berberine), owing to their promising cellular effect on the glycolysis-ApoE nexus, warrant further investigation in suitable in vivo models of AD.

## 1. Introduction

Alzheimer’s disease (AD) is a progressive, age-associated disease, which is clinically characterized by extensive memory loss and severe impairment of various other cognitive functions [1]. Two major pathological characteristics of AD include extracellular deposits of amyloid beta (Aβ) protein and intracellular accumulation of neurofibrillary tangles of tau protein [2,3,4]. Numerous studies, including molecular, cellular, and animal models, imply a central role of Aβ protein, specifically as small oligomers, in the pathogenesis and progression of AD [5,6]. The abnormal Aβ deposits in the AD brain are known to initiate a deleterious cascade of events involving the formation of neurofibrillary tangles of tau, reactive gliosis, activated microglia and complement pathways, severe inflammation, early synapse loss, and neurotransmitter deficit [2,6,7]. In this context, two anti-Aβ monoclonal antibodies have recently received accelerated approvals by the United States Food and Drug Administration (FDA), viz. aducanumab (June 2021), and lecanemab (January 2023), with the latter receiving full FDA approval in July 2023 owing to its compelling clinical data [8,9].

The pathological Aβ peptides are generated by the sequential proteolytic cleavage of amyloid precursor protein (APP) by β- and γ-secretase [10]. Thus, in addition to the approved anti-Aβ antibodies, there has been a significant interest in developing β- and γ-secretase inhibitors with the aim of lowering Aβ production. Alternatively, age is the most significant risk factor for AD, and the brain’s ability to clear Aβ is shown to diminish with age in mice [11,12]. Indeed, a real-time Aβ turnover study showed Aβ production to be normal, while Aβ clearance was significantly decreased in AD patients compared to healthy controls [13]. Notably, the rates of Aβ synthesis and clearance were shown to be similar in normal subjects [14]. Therefore, decreased clearance of Aβ leading to its increased deposition in the brain may play a predominant role in AD pathogenesis, and thus, molecular mechanisms underlying decreased Aβ clearance in an AD brain may provide novel therapeutic targets with potentially disease-modifying effects.

It is noteworthy here that, in addition to the pathophysiological changes including Aβ deposition, AD is also characterized by abnormal metabolic changes. Among these, declined cerebral glucose metabolism is one of the distinct and earliest characteristics of the AD brain [15] and is clearly associated with the rapid decline in cognitive abilities of AD patients [16]. Indeed, the ATP production from brain glucose utilization is significantly decreased by ~50% in sporadic AD compared with healthy controls [17]. The glucose metabolism is also significantly decreased in patients with mild cognitive impairment (MCI) who subsequently develop AD [18]. Furthermore, in subjects that are genetically predisposed to developing AD, cerebral metabolic changes occur well before the manifestation of any pathophysiological signs of the disease [19]. Thus, abnormal glucose metabolism is a distinct and early event in AD pathology, which may play a central role in the manifestation of AD-associated neuropathological changes, including enhanced Aβ deposition [20,21]. It is noteworthy here that Aβ is deposited mainly in those AD brain areas that are known to rely primarily upon glycolysis [22]. Thus, abnormal glucose metabolism in the AD-affected brain regions may lead to the loss of critical glucose-dependent functions, which, in turn, may result in increased Aβ deposition in those brain regions. Despite these accumulating data, however, the molecular mechanism underlying the potential causal relationship between glucose hypometabolism and decreased Aβ clearance leading to its increased deposition in AD is not clear.

In this context, apolipoprotein E (ApoE), the most significant risk factor for late-onset AD [23], is known to play a critical role in Aβ clearance from the brain [24]. Notably, the Aβ-clearing effect of ApoE is found to be isoform-specific (ApoE2 > ApoE3 > ApoE4) [25]. Indeed, the Aβ levels and amyloid plaque loads are found to be higher in ApoE4 carrier brains than ApoE3 and ApoE2 genotypes, with the lowest levels in ApoE2 carrier brains [26,27]. Accordingly, ApoE4 has been shown to confer a significantly higher risk for late-onset AD compared to both ApoE3 and ApoE2 genotypes. Importantly, the observed ApoE isoform-specific effect on Aβ clearance is attributed to its lipidation status; increasing the lipidation status of ApoE3 and ApoE4 is shown to attenuate the ApoE isoform-specific differences in Aβ clearance [25]. The lipidation of ApoE promotes Aβ degradation through multiple pathways, including enhancing the ability of insulin-degrading enzyme (IDE) to degrade extracellular Aβ [23] or uptake and intracellular degradation by the glial lysosomal system [28]. The different ApoE isoforms may also compete differentially with soluble Aβ for uptake by astrocytes through the low-density lipoprotein receptor-related protein 1 (LRP1) mechanism [29], which is also involved in peripheral clearance of Aβ across the blood–brain barrier [30]. The observed differential binding with LRP1 may be attributed to the inherent structural differences of ApoE isoforms, making ApoE4 a less stable and an inferior lipid carrier compared to ApoE2 and ApoE3 [31,32,33,34,35]. Taken together, the highly lipidated ApoE2 or ApoE3 isoforms significantly enhance Aβ clearance from the brain, while poorly lipidated ApoE4 may lead to the formation of Aβ fibrils and Aβ deposition in an AD brain [36,37]. It is noteworthy that the different ApoE isoforms with differential lipidation status may also play a role beyond Aβ pathology, such as tau hyperphosphorylation [38,39,40], neuroinflammation [41], impaired neurogenesis [42], abnormal dendritic spine density [43], and mitochondrial dysfunction and neurotoxicity [44,45].

Here, it is noteworthy that the activation of the liver x receptors (LXRs) has been shown previously to significantly increase ApoE lipidation status [46], and glucose, and glycolytic intermediates have been shown to be effective endogenous LXR agonists that directly bind and activate the LXRs [47]. Based on these accumulating data we hypothesized that the decreased cerebral glucose metabolism, which is an early and distinct characteristic of AD, may lead to decreased LXR activation and poor ApoE lipidation, which in turn may be responsible for decreased Aβ clearance, and, hence, its increased deposition in the AD brain [20]. Therefore, our present study focused on investigating such a potential relationship between glucose metabolism and ApoE lipidation in astrocytes from human ApoE4 knock-in mice. The ApoE-glycolysis mechanistic knowledge thus obtained from this cellular Alzheimer’s model was further utilized to discover novel glycolytic enhancers for enhancing ApoE lipidation, with potential applications against AD pathology.

## 2. Results and Discussion

### 2.1. Dose-Dependent Effect of Glucose on ABCA1/G1 Expression and ApoE Lipidation

In the early stages of AD pathology, astrocytes have been shown to increase glucose metabolism, possibly implying a compensatory response to the disease stress [48]. This is further supported by a recent ground-breaking proteomic study in AD patients [49] that revealed a significant increase in glycolysis proteins decades before the disease onset, possibly as a protective metabolic boost. Indeed, ApoE4 is strongly associated with decreased cerebral glucose metabolism in both AD patients and nondemented individuals [50,51].

Therefore, to investigate the potential role of astrocytic glucose metabolism in ApoE lipidation, the immortalized astrocytes from human ApoE4 knock-in mice were cultured in serum-free media containing different concentrations of glucose, ranging from low 1 g/L (5.55 mM) to high 4.5 g/L (25 mM). Our present study specifically utilized astrocytes from human ApoE4 knock-in mice, as ApoE is mainly derived from astrocytes in the normal human brain at both mRNA [52] and protein [53] levels. Although microglial ApoE expression is induced in an AD mice model [52], only a small fraction of microglia in human AD brains show ApoE immunoreactivity [54]. Also, although neurons do not express ApoE under normal conditions; ApoE is found to be produced in neurons under conditions of stress or aging [38,55]. Accordingly, neuronal ApoE expression has been linked to the major hallmarks of AD, including tau pathology and selective neurodegeneration [56]. Nevertheless, our present study mainly focused on the astrocytes being the major source of human-brain ApoE.

**Figure 1:**
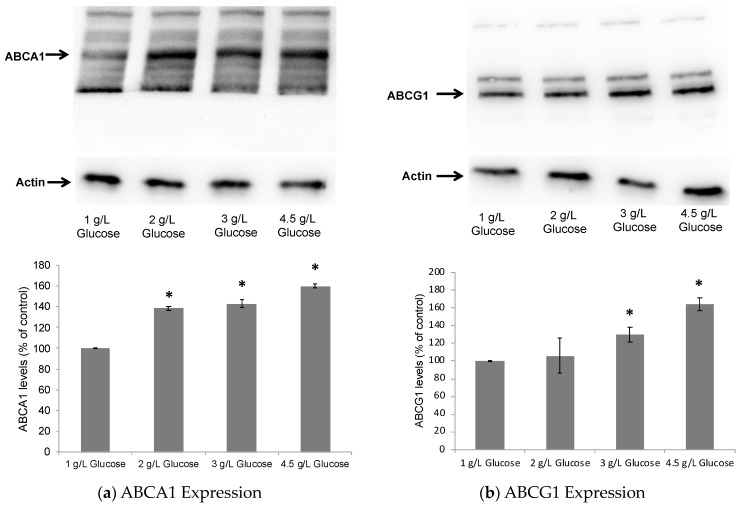
Dose-dependent effect of glucose on ABCA1 and ABCG1 levels in astrocytes. Immortalized mouse astrocytes expressing human ApoE4 were treated with 1 g/L, 2 g/L, 3 g/L or 4.5 g/L of D-glucose for 24 h. Immunoblots show significant upregulation of (**a**) ABCA1 and (**b**) ABCG1 in ApoE4-expressing cells in a dose-dependent manner. Data represent mean ± SD of three experiments. (One-way ANOVA with Tukey’s post hoc method—*, *p* < 0.05 compared with 1 g/L glucose condition).

The primary function of ApoE is to traffic lipids in the brain, and its lipidation is achieved mainly through the action of the ATP-binding cassette transporters, viz. ATP-binding cassette subfamily A member 1 (ABCA1) and subfamily G member 1 (ABCG1) [23]. The expression of ApoE and ABCA1/G1 is regulated by LXRs, and pharmacological activation of LXRs has been shown to induce a significant increase in ApoE lipidation and secretion by astrocytes [41]. Therefore, after 24 h of treatment with glucose, the astrocytes were lysed, and Western blot analyses were performed to determine the cellular levels of ABCA1 as an indicator of potential LXR activation [57]. As seen in Figure 1, glucose significantly increased LXR activation in a dose-dependent manner, as evident by upregulation in ABCA1/G1 levels in ApoE4-expressing cells.

**Figure 2:**
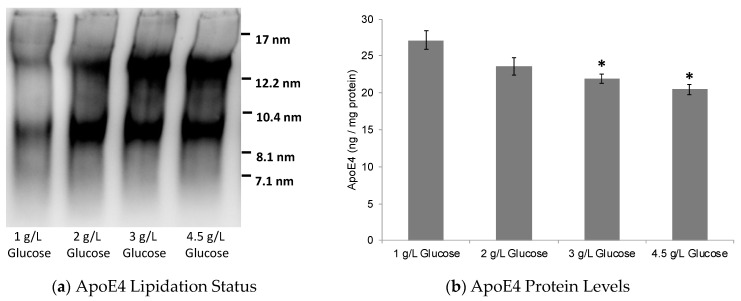
Effect of glucose on the levels and lipidation status of ApoE secreted by astrocytes. The ApoE4-expressing astrocytes were treated with 1 g/L, 2 g/L, 3 g/L, or 4.5 g/L D-glucose for 24 h. (**a**) The native blots on culture media show significant increase in lipidated ApoE with increasing glucose concentration. (**b**) The ApoE ELISA showed a slight, but statistically significant, decrease in ApoE4 protein levels. Data represent mean ± SD of three experiments. (One-way ANOVA with Tukey’s post hoc method—*, *p* < 0.05 compared with 1 g/L glucose condition).

Here, it is noteworthy that glucose as well as glycolytic intermediates have been previously shown to directly bind and activate the LXRs in HepG2 cells [46]. This further supports the glucose-induced LXR activation in astrocytes observed in this study. In accordance with this LXR activation, the possible increase in ApoE secretion in the cell culture media and the ApoE lipidation status were tested using an enzyme-linked immunosorbent assay (ELISA) and native gel electrophoresis, respectively. As expected, ApoE lipidation was significantly enhanced with glucose treatment in ApoE4-expressing cells (Figure 2). The ApoE levels, however, were slightly decreased with an increasing glucose concentration in the culture medium (Figure 2).

Here, it is interesting to note that decreasing the level of ApoE4 protein has been shown to significantly lower amyloid plaque deposition in transgenic mice models [58]. More importantly, two human subjects with ApoE4 loss-of-function variants were recently shown to be completely amyloid plaque-free and cognitively sharp, strongly supporting the knockdown of ApoE4 as a promising therapeutic strategy against AD [59]. These studies, together with our present data, may suggest that the observed glucose-induced upregulation in ApoE lipidation, together with a potential decrease in ApoE protein levels, may prove to be beneficial in terms of potential Aβ clearance as well as against other Aβ-independent AD pathways.

### 2.2. Glycolytic Control of LXR-Induced ABCA1/G1 Expression and ApoE Lipidation

**Figure 3:**
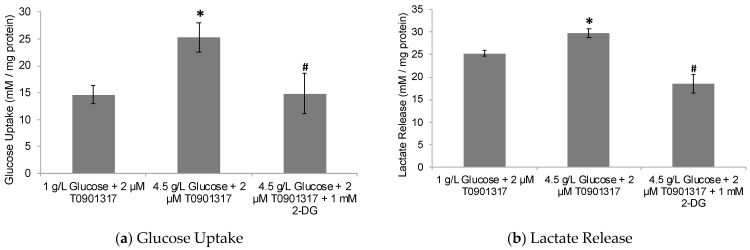
Glucose-metabolism analysis in ApoE4-expressing astrocytes. The cells were treated for 24 h with 2 µM T0901317, an LXR agonist, in presence of low-glucose (1 g/L) and high-glucose (4.5 g/L) media, with the latter also in the presence of 1 mM 2-DG. (**a**) Glucose uptake and (**b**) lactate production in 24 h period. Data represent mean ± SD of three experiments. (One-way ANOVA with Tukey’s post hoc method—*, *p* < 0.05 compared with low-glucose condition and #, *p* < 0.05 compared with high-glucose condition).

To further investigate the potential role of glucose metabolism in LXR activation leading to ApoE lipidation and secretion by astrocytes, the human ApoE4-expressing astrocytes were treated with a potent LXR activator, T0901317, in low-glucose (1 g/L) and high-glucose (4.5 g/L) treatment media. The goal was to determine the ApoE lipidation and secretion status in the conditioned media from these co-treated astrocytes compared to untreated control ones. The glucose uptake and lactate production were found to be significantly higher in the human ApoE4-expressing astrocytes treated with high-glucose media compared to low-glucose media (Figure 3).

**Figure 4:**
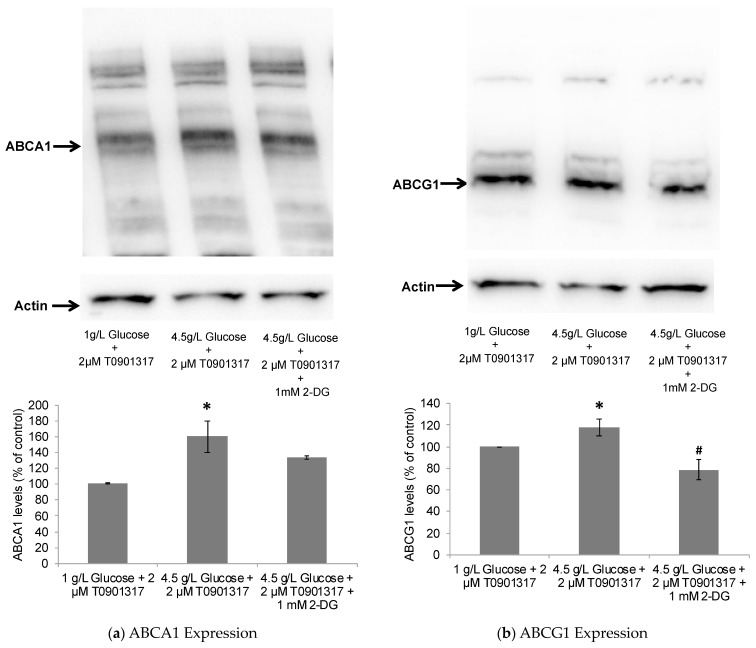
Effect of glucose on T0901317-induced ABCA1/G1 expression in astrocytes. The human ApoE4-expressing astrocytes cultured in low-glucose (1 g/L) and high-glucose (4.5 g/L) media were treated for 24 h with 2 µM T0901317. Immunoblots show significant upregulation of (**a**) ABCA1 and (**b**) ABCG1 in ApoE4-expressing cells in a glucose-dependent manner. The T0901317-induced ABCA1/G1 upregulation was downregulated by glycolytic inhibition with 1 mM 2-DG. Data represent mean ± SD of three experiments. (One-way ANOVA with Tukey’s post hoc method—*, *p* < 0.05 compared with low-glucose condition and #, *p* < 0.05 compared with high-glucose condition).

In accordance with the observed enhanced glycolysis, the LXR activation by T0901317, as indicated by ABCA1 and ABCG1 upregulation, was also found to be significantly higher in the presence of high-glucose versus low-glucose media (Figure 4). Here, it is noteworthy that the LXR activation with another potent pharmacological LXR agonist (GW3965) has also been previously shown to be dependent upon the presence, uptake, and metabolism of glucose by HepG2 cells [46].

**Figure 5:**
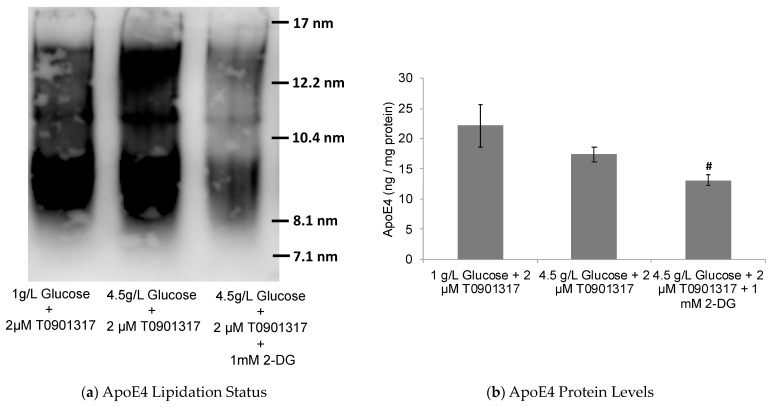
Glucose-dependent ApoE lipidation induced by T0901317 in astrocytes. The human ApoE4-expressing astrocytes cultured in low-glucose (1 g/L) and high-glucose (4.5 g/L) media were treated for 24 h with 2 µM T0901317. (**a**) The native blots show T0901317-induced significant increase in lipidated ApoE in a glucose-dependent manner, which was effectively inhibited in presence of the glycolytic inhibitor 2-DG. (**b**) The levels of the secreted ApoE4 protein were also decreased in the presence of 2-DG in the ApoE4-expressing cells. Data represent mean ± SD of three experiments. (One-way ANOVA with Tukey’s post hoc method—#, *p* < 0.05 compared with high-glucose condition).

Furthermore, ApoE lipidation was also significantly enhanced in cells treated with T0901317 in high-glucose compared to low-glucose media (Figure 5). On the other hand, the levels of secreted ApoE4 were found to be slightly decreased with an increased glucose concentration and its metabolism (Figure 5). These data further support our earlier results emphasizing the role of glucose metabolism in LXR activation, leading, in turn, to ABCA1/G1 upregulation and ApoE lipidation.

Taken together, these data may indicate that ApoE4-expressing astrocytes are sensitive to glycolytic effects on the LXR-ApoE regulatory pathway. Here, it is noteworthy that cognitively healthy ApoE4 carriers are known to show abnormalities in brain glucose metabolism before any Aβ pathology is detected [60]. Thus, ApoE4 carriers may be inherently susceptible to poor ApoE lipidation due to glucose hypometabolism and, hence, increased Aβ deposition, leading to an increased risk for AD pathogenesis. To further investigate this notion, the human ApoE4-expressing astrocytes were subjected to treatment with 2-deoxyglucose (2-DG), a glycolytic inhibitor. The treatment of ApoE4-expressing astrocytes with 2-DG significantly inhibited cellular glycolysis, as indicated by decreased glucose uptake and lactate production by the cells (Figure 3). The observed glycolytic inhibition by 2-DG in ApoE4-expressing cells was further accompanied by the downregulation in ABCA1/G1 levels (Figure 4), together with the decreased ApoE4 lipidation (Figure 5).

### 2.3. Discovery of Bioactive Compounds for Enhanced Astrocytic Glycolysis

Glucose metabolism is significantly affected in AD compared to healthy controls [61], and abnormal aerobic glycolysis may play a central role in the observed Aβ deposition and other disease abnormalities observed in the AD-affected regions through the potential effect on the ApoE pathway. Our present results support this potential ‘glycolysis-ApoE-AD’ nexus. Thus, pharmacological agents that can target cellular energy metabolism, specifically aerobic glycolysis, may hold significant therapeutic potential against AD. In this context, several FDA-approved drugs and other bioactive molecules have been previously shown to possess the ability to significantly shift cellular metabolism towards aerobic glycolysis [62] and also protect neurons from glucose toxicity [63].

Therefore, we planned to treat astrocytes from human ApoE4 knock-in mice with a select set of FDA-approved drugs and other bioactive molecules (total of 20) for their potential glycolytic enhancing activity in these cells. These drugs were selected based on the published literature indicating their potential role in modulating cellular glucose metabolism, not necessarily in astrocytes [62,63]. Thus, our primary goal was to determine which of these molecules positively modulate glucose uptake and lactate production, specifically in human ApoE4-expressing astrocytes. The test drugs involved β-blockers, nootropics, anti-diabetics, anti-cancer agents, anti-histamines, anti-psychotics, and non-steroidal anti-inflammatory drugs. Note that the selected anti-diabetics are not PPARγ (peroxisome proliferator-activated receptor gamma) agonists (e.g., rosiglitazone, pioglitazone, etc.), as PPARγ is known to directly bind and activate LXR, leading to ApoE lipidation, while our present study aims to discover novel enhancers of ApoE lipidation and secretion through glycolytic stimulation in astrocytes.

**Figure 6:**
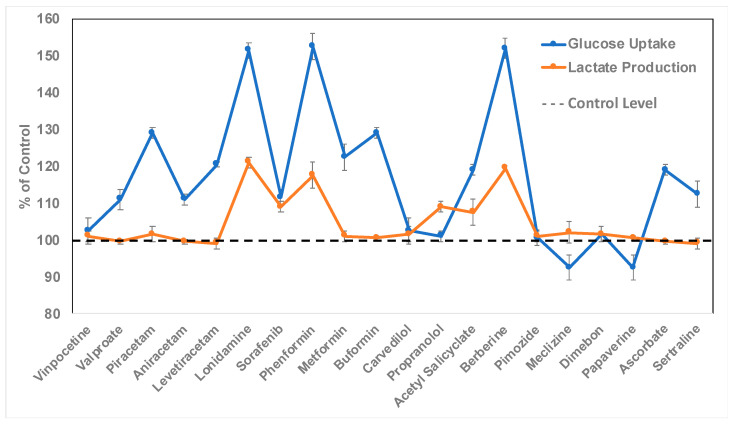
Pharmacological effect on astrocytic glucose uptake and lactate production. The human ApoE4-expressing astrocytes were treated for 24 h with 5 or 10 µM of respective drug molecules in presence of high-glucose (4.5 g/L) media. Three drug molecules (lonidamine, phenformin, and berberine) out of a total of 20 tested showed significantly enhanced glucose uptake and lactate production by ApoE4-expressing astrocytes.

Accordingly, astrocytes were treated at four different concentrations (1, 2, 5, and 10 μM) of each of the 20 drugs. After 24 h of treatment, the cells were inspected to determine the highest concentration for each drug at which there was no effect on cell viability and morphology. This concentration was 10 μM for 17 out of 20 drugs, and 5 μM for the remaining 3 drugs (valproate, buformin, and sertraline). Therefore, astrocytes were treated at the highest concentration of either 5 or 10 μM of respective drugs for 24 h in high-glucose media (4.5 g/L). Three drug molecules (lonidamine, phenformin, and berberine) were found to significantly enhance glucose uptake and lactate production by astrocytes (Figure 6). It is noteworthy here that lonidamine, an anti-cancer agent, is an inhibitor of mitochondrial hexokinase known to suppress aerobic glycolysis in cancer cells [64,65], but to enhance it in normal cells [66]. The anti-diabetic drug phenformin is from the biguanide class of drugs, which are known to activate AMPK (AMP-activated protein kinase) and enhance glycolysis [67,68]. Interestingly, phenformin was shown to be superior in enhancing astrocytic glycolysis compared to the other two biguanide drugs, viz. metformin and buformin. Finally, berberine is a natural bioactive molecule with a variety of therapeutic effects, including anti-diabetic activity comparable to biguanide drugs [69].

### 2.4. Effect of Glycolytic Stimulants on ApoE Lipidation and Secretion by Astrocytes

**Figure 7:**
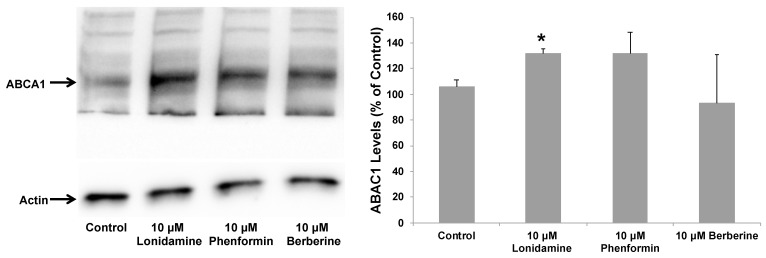
Effect of glycolytic enhancer drugs on ABCA1 levels in astrocytes. Immortalized mouse astrocytes expressing human ApoE4 were treated with 10 μM lonidamine, phenformin, or berberine for 24 h. Data represent mean ± SD of three experiments. (One-way ANOVA with Tukey’s post hoc method—*, *p* < 0.05 compared with untreated controls).

Our present data described above suggested that increasing glycolysis may lead to ApoE lipidation, in addition to affecting its secretion by astrocytes. Therefore, we planned to treat astrocytes from human ApoE4 knock-in mice with the three drug molecules that we found to exhibit the glycolytic enhancing ability (lonidamine, phenformin, and berberine). To investigate if these glycolytic enhancers stimulate LXR activation leading to ApoE lipidation and secretion by astrocytes, the human ApoE4-expressing astrocytes were cultured for 24 h in normal, high-glucose (4.5 g/L) treatment media containing lonidamine, phenformin, or berberine. Lonidamine and phenformin enhanced LXR activation, as indicated by ABCA1 upregulation in the ApoE4-expressing cells (Figure 7).

**Figure 8:**
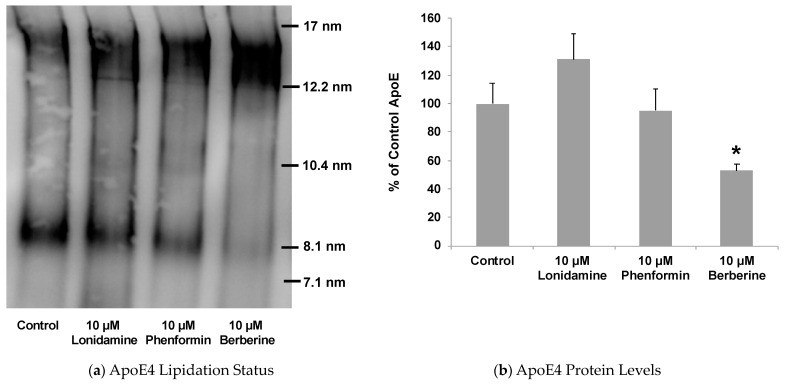
Effect of glycolytic enhancer drugs on the levels and lipidation status of ApoE secreted by astrocytes. The ApoE4-expressing astrocytes were treated with 10 μM lonidamine, phenformin, or berberine for 24 h. The native blots on culture media show significant increase in lipidated ApoE due to drug treatment. The ApoE ELISA showed significant decrease in ApoE4 levels due to berberine treatment. Data represent mean ± SD of three experiments. (One-way ANOVA with Tukey’s post hoc method—*, *p* < 0.05 compared with untreated controls).

In line with their observed effect on LXR activation, lonidamine and phenformin caused significant enhancement in ApoE lipidation (Figure 8). Berberine, on the other hand, showed a slight decrease in ABCA1 levels, which did not reach statistical significance (Figure 7). Nonetheless, berberine also caused significant enhancement in ApoE lipidation (Figure 8). Thus, unlike lonidamine and phenformin which were found to activate the LXR pathway, as evident by ABCA1 upregulation, the berberine effect on ApoE lipidation appears to be LXR-independent. Such an LXR-independent effect on astrocytic ApoE has been observed with class I histone deacetylase (HDAC) inhibitors [70], a chrysanthemic ester molecule [71], and a direct peptide activator of ABCA1 (CS-6253) [72]. Such LXR-independent enhancers of ApoE lipidation would be highly selective toward AD, as they may be void of broad activation of other nuclear hormone receptor targets and, thus, not cause the known toxicity issues associated with LXR agonists, including fatty liver and increased plasma triglyceride levels [73]. Indeed, a recent study showed the LXR agonist GW3965 to significantly upregulate ABCA1 and mitigate tauopathy and neuroinflammation in mice but is also known to cause liver damage through fatty acid accumulation [74]. Thus, the potential LXR-independent mechanism behind berberine-induced ApoE lipidation warrants further investigation.

It is also noteworthy here that lonidamine has been shown to inhibit glycolysis in microglia from the AD model, in turn making them similar to the microglia from wild-type mice in terms of enhanced phagocytosis, increased lysosomal degradation of internalized Aβ, and decreased secretion of pro-inflammatory cytokines [75]. Taken together with our present data, lonidamine may have a dual beneficial effect against AD, through both enhanced ApoE lipidation and rescuing of microglial defects associated with AD. Note that lonidamine is shown to attenuate inflammatory injury by blocking diverse types of inflammasome activation independent of its known targets, including hexokinase 2 (HK2) [76]. Our present study shows yet another mechanism through which lonidamine may help against AD pathology, whether through HK2-dependent or -independent pathways remains to be seen.

## 3. Materials and Methods

### 3.1. Cell Culture and Treatment

The human ApoE4-expressing astrocytes [77] were obtained from Dr. David M. Holtzman (Washington University Medical School, St. Louis, MO, USA). The astrocytes were cultured in 75 cm^2^ tissue-culture flasks in a growth medium containing Dulbecco’s Modified Eagle’s Medium (DMEM) supplemented with 10% Fetal Bovine Serum, 1 mM sodium pyruvate, and 200 µg/mL genticin (all cell culture supplies were from Life Technologies, Carlsbad, CA, USA). To carry out the experiments, cells were plated in 12-well tissue-culture plates and grown to confluence in a growth medium for 24 h. After 24 h, the growth medium was replaced with a serum-free treatment medium containing DMEM-F12 w/HEPES supplemented with 1 mM sodium pyruvate and N-2 neuronal cell supplement.

### 3.2. Glucose-Metabolism Studies

To measure the uptake of glucose and consequent release of lactate, the astrocytic cell-culture media were collected immediately after 24 h of experimental treatment and centrifuged for 15 min at 3000× *g* to remove any cell debris. The glucose and lactate concentrations in the conditioned media were measured by using enzymatic glucose (Stanbio Laboratories, Boerne, TX, USA) and lactate (Trinity Biotech, Jamestown, NY, USA) assays. The glucose uptake and lactate production values were calculated by using the differences between their concentrations in the media before and after the 24 h treatment. The data were then normalized by using the total intracellular protein levels.

### 3.3. Western Blotting Analysis

The cells were washed three times with ice-cold PBS and lysed for 20 min by scraping into ice-cold lysis buffer (20 mM Tris-HCl, pH 7.5, 150 mM NaCl, 1 mM EDTA, 1% Nonidet P-40, 0.5% deoxycholate, 0.1% SDS, and protease and phosphatase inhibitors, all chemicals from Sigma, St. Louis, MO, USA) [48]. The total protein concentration in cell lysate was measured by a Bicinchoninic acid protein assay kit (Pierce Biotechnology, Waltham, MA, USA) and equal amounts of total protein were run on 7.5% SDS-PAGE gels (BioRad, Hercules, CA, USA), followed by transfer to nitrocellulose membranes for 2.5 h at 100 V. The membranes were then probed with the appropriate primary antibodies [1:1000 ABCA1 (Millipore, Burlington, MA, USA) and 1:2000 actin (Sigma, MO)] and imaged with the BioRad ChemiDoc XRS+ using the Pierce SuperSignal West Femto Maximum Sensitivity Substrate (Pierce Biotechnology).

### 3.4. ApoE Native Gel Electrophoresis and ELISA

The cell-culture media were collected on ice after experimental treatment and centrifuged for 15 min at 3000× *g* to remove any cell debris. The equal volume of the clear supernatant from each condition was then transferred to Amicon Ultra-4 centrifugal filter units with MWCO of 10 kDa (Millipore) and centrifuged at 3000× *g* to concentrate the media by ~30X. The concentrated media were then subjected to native gel electrophoresis and ELISA to determine the ApoE lipidation status and total ApoE protein levels, respectively. For native gel electrophoresis, an equal amount of concentrated media was run on 7.5% Tris-glycine native gels (BioRad), followed by transfer to nitrocellulose membranes for 2.5 h at 100 V. The membranes were incubated at 4 °C overnight with primary antibody [1:500 ApoE (Academy BioMedical, Houston, TX, USA, cat#50A-G1)] and imaged with the BioRad ChemiDoc XRS+. The size of the ApoE particles was determined by comparison to a native size marker (Amersham Biosciences, Piscataway, NJ, USA). The ApoE ELISA was carried out using a human ApoE ELISA kit (Abcam, Waltham, MA, USA) according to the manufacturer’s instructions.

### 3.5. Statistical Analysis

The data are expressed as the mean ± SD for three independent experiments. Student’s *t*-test and one-way ANOVA with Tukey’s post hoc method in GraphPad Prism 10.1.2 were used to evaluate the statistical significance between different treatment groups. A value of *p* < 0.05 indicates a significant difference.

## 4. Conclusions

The present study explored the potential synergistic relationship between glucose metabolism and ApoE lipidation, leading to the discovery of novel glycolytic stimulants against AD. Specifically, a central role of astrocytic glucose metabolism was observed in basal as well LXR agonist-induced ABCA1/G1 upregulation and subsequent enhancement in ApoE lipidation status. In particular, a higher glucose concentration led to increased ApoE lipidation, while inhibition of glycolysis significantly downregulated it. The observed sensitivity of ApoE4-expressing astrocytes to glycolytic effects on the LXR-ApoE regulatory pathway may imply the potential causal link between abnormal glucose metabolism and ApoE-related pathways in AD. From a therapeutic point of view, it may also suggest that elevating astrocytic glucose metabolism may lead to enhanced ApoE lipidation and a consequent Aβ degradation with disease-modifying effects against AD. In support, the GAB2 (GRB2-associated binding protein 2) gene haplotype is shown to be protective against AD in even highly susceptible ApoE4 carriers and is associated with higher glucose metabolism in AD-affected brain regions [78]. Therefore, to identify potential therapeutic strategies against AD, we explored several bioactive compounds including approved and investigational drugs for their potential effects on astrocytic glycolysis. Three bioactive compounds (lonidamine, phenformin, and berberine) were found to significantly stimulate glucose uptake and lactate production in astrocytes, establishing their glycolytic-enhancing properties. Importantly, all three compounds demonstrated the ability to significantly enhance ApoE lipidation, suggesting a promising avenue for modulating the glycolysis-ApoE-AD nexus through both increased clearance and decreased deposition of Aβ peptides.

In conclusion, our study sheds light on the intricate relationship between glucose metabolism and ApoE regulation in astrocytes, emphasizing the potential role of glycolysis in modulating AD-related pathways. Our newly identified glycolytic enhancers (lonidamine, phenformin, and berberine) show promise as candidates for further investigation in developing novel therapeutic strategies against AD by targeting astrocytic glucose metabolism and ApoE regulation. These findings contribute valuable insights into the molecular mechanisms underlying AD pathogenesis and open avenues for future research in the quest for effective AD treatments.

## Data Availability

The data are available on request from the corresponding author.
